# Cardiac tissue slices: preparation, handling, and successful optical mapping

**DOI:** 10.1152/ajpheart.00556.2014

**Published:** 2015-01-16

**Authors:** Ken Wang, Peter Lee, Gary R. Mirams, Padmini Sarathchandra, Thomas K. Borg, David J. Gavaghan, Peter Kohl, Christian Bollensdorff

**Affiliations:** ^1^Department of Computer Science, University of Oxford, Oxford, United Kingdom;; ^2^Department of Physics, University of Oxford, Clarendon Laboratory, Oxford, United Kingdom;; ^3^Harefield Heart Science Centre, National Heart and Lung Institute, Imperial College London, Middlesex, United Kingdom;; ^4^Department of Regenerative Medicine and Cell Biology, University of South Carolina School of Medicine, Charleston, South Carolina; and; ^5^Qatar Cardiovascular Research Center, Qatar Foundation, Doha, Qatar

**Keywords:** heart, optical mapping, multiparametric, high spatial resolution, voltage- and calcium-sensitive dyes, live tissue slices

## Abstract

Cardiac tissue slices are becoming increasingly popular as a model system for cardiac electrophysiology and pharmacology research and development. Here, we describe in detail the preparation, handling, and optical mapping of transmembrane potential and intracellular free calcium concentration transients (CaT) in ventricular tissue slices from guinea pigs and rabbits. Slices cut in the epicardium-tangential plane contained well-aligned in-slice myocardial cell strands (“fibers”) in subepicardial and midmyocardial sections. Cut with a high-precision slow-advancing microtome at a thickness of 350 to 400 μm, tissue slices preserved essential action potential (AP) properties of the precutting Langendorff-perfused heart. We identified the need for a postcutting recovery period of 36 min (guinea pig) and 63 min (rabbit) to reach 97.5% of final steady-state values for AP duration (APD) (identified by exponential fitting). There was no significant difference between the postcutting recovery dynamics in slices obtained using 2,3-butanedione 2-monoxime or blebistatin as electromechanical uncouplers during the cutting process. A rapid increase in APD, seen after cutting, was caused by exposure to ice-cold solution during the slicing procedure, not by tissue injury, differences in uncouplers, or pH-buffers (bicarbonate; HEPES). To characterize intrinsic patterns of CaT, AP, and conduction, a combination of multipoint and field stimulation should be used to avoid misinterpretation based on source-sink effects. In summary, we describe in detail the preparation, mapping, and data analysis approaches for reproducible cardiac tissue slice-based investigations into AP and CaT dynamics.

live tissue slices are well-established pseudo-two-dimensional (2D) models for research into organ (patho-)physiology and drug effects, in particular for brain (54) and liver (29). In cardiac research, tissue slices have received less attention, compared with models such as the Langendorff-perfused whole heart, arterially perfused tissue wedges, cell cultures, or isolated single cells (21). Nonetheless, cardiac tissue slices represent a unique model for cardiac electrophysiology (20) and also open up new possibilities for pharmacological research and development (19, 21). Cardiac tissue slices permit the observation of different functional parameters such as transmembrane potential (*V*_m_) and calcium transients (CaT) and their correlation to cellular substrates, which is often more difficult in three-dimensional (3D) model systems. Compared with single isolated cells or cell culture, slices benefit from inclusion of the various cell types that make up native tissue such as myocytes and fibroblasts in myocardium (12). The locally preserved cell-cell electrical and mechanical connections (39, 72) and the extracellular matrix reflect a more in vivo like profile than other 2D or lower dimensionality models (13).

The use cardiac tissue slices in metabolism research dates back at least eight decades to the work by Pincus (58) and Thienes et al. (70, 71). This technology has been applied to the measurement of oxygen consumption (66, 74), assessment of ATP-sensitive potassium channel (K_ATP_) contributions to ischemic preconditioning (34), electrophysiological studies of reentry induction by premature stimuli (56), and drug testing (9, 10).

Numerous technical challenges must be addressed to obtain consistent vital cardiac slices with uniform thickness and minimal tissue damage, including slow vibratome blade advance speeds (50 μm/s or less), minimal z-axis blade deflection (<1 μm), slice thicknesses, and alignment of the cutting plane. In previous reports, cardiac slice thicknesses varied from 150 (33) to 500 μm (20, 56). To maintain sufficient oxygen diffusion to all cells inside the section, investigators now favor slices of no more than 400 μm thickness (3), to avoid potential exposure to hypoxic conditions. As suggested by Yashura et al. in the 1990s (74), cutting ventricular tissue tangentially to the epicardial surface allows for an optimized alignment of (at least near-epicardial) slices with locally prevailing cell orientation, compared with transmurally directed sections. This was confirmed by Bussek et al. (10), assessing the results of different cutting directions using two-photon microscopy and histology. Previous studies used different recovery protocols, with durations ranging from 30 min (7) to 2 h (74) and with different buffer solutions. No detailed information about the influence of postcutting recovery protocols on electrophysiological measurements has been published to date.

Most published electrophysiology studies on cardiac tissue slices have obtained relatively low spatial resolution data, from point-recordings by patch clamp (7) or sharp electrodes (6, 8, 33) to 60 channel multielectrode arrays (10, 11). Few studies (20, 45, 56) have used high spatial resolution optical methods to monitor electrophysiological parameters. This includes our proof-of-principle-studies (45, 69) in which we employed dual *V*_m_ and CaT mapping of ventricular slices to illustrate the suitability of the approach for studying the effects of mechanoelectric feedback and for dynamic monitoring of drug effects. Different camera types have been used for optical mapping of *V*_m_ and CaT, most commonly CMOS (28, 41, 63) and EMCCD cameras (43). In cardiac tissue slices, the signal is strong enough to be successfully recorded by cost-effective EMCCD cameras. For more detailed reviews on cardiac optical mapping approaches, please refer to articles by Herron et al. (35) and by Entcheva and Bien (23). Cameras with >10,000 pixels and with a frame rate in the kHz-range acquire enormous amounts of data. Processing and analyzing of these data can be challenging. Freely available programs like ImageJ (27, 61) are helpful, and more sophisticated methods have been developed for processing optical imaging data obtained from single cells and whole heart (42, 67, 75).

The aim of this study was to refine experimental conditions for successful tissue slice preparation, optical mapping, and data analysis. Attention is drawn to recovery times, temperature effects, and buffer solution composition. We describe an approach for semiautomated data analysis, which achieves reliable extraction of information on action potential (AP) and CaT parameters. The utility of these recommendations is illustrated using recordings from rabbit and guinea pig cardiac tissue slices.

## MATERIALS AND METHODS

### Heart Isolation

Male New Zealand White rabbits (1–2 kg, *n* = 9) and female guinea pigs (250–400 g, *n* = 3) were humanely killed after local ethical approval, either by anesthetic overdose (pentobarbital, 70 mg/kg for rabbits) or cervical dislocation (guinea pigs), in accordance with Schedule 1 of the UK Home Office Animals (Scientific Procedures) Act 1986. Hearts were quickly excised and perfused in Langendorff-mode with bicarbonate-buffered solution (containing in mmol/l: 123 NaCl, 1.8 CaCl_2_, 5.4 KCl, 1.2 MgCl_2_, 1.4 NaH_2_PO_4_, 24 NaHCO_3_, and 10 glucose; bubbled with 95% O_2_-5% CO_2_; pH 7.4 at 35 ± 2°C). For electromechanical uncoupling, blebbistatin (10 μmol/l); Ascent Scientific, Cambridge, UK) was added after dye loading and before slicing/optical mapping. All chemicals were obtained from Sigma-Aldrich (Dorset, UK), unless otherwise stated. Note that fat accumulation at the epicardial surface increases with animal age. Fat tissue is difficult to cut and can blunt the blade, so we recommend careful manual removal of fat tissue before slicing.

### Dye Loading

#### Rabbit.

Fluorescent dyes were loaded via the coronary circulation, applied by injection into the aortic cannula. First, 22 μl of a solution containing the voltage-sensitive dye di-4-ANBDQPQ (20 μl of stock solution 27 mmol/l in ethanol; University of Connecticut Health Center) and Pluronic F-127 (2 μl of a 20% stock solution in DMSO; Life Technologies, Paisley, UK) were slowly added over a 4- to 5-min period (i.e., at a Langendorff perfusion rate of 16–20 ml/min, the dye was diluted in ∼65–100 ml bicarbonate-buffered solution during application). To improve calcium dye loading and retention of the dye in the cytoplasmic matrix, rabbit hearts were preperfused with bicarbonate-buffered solution containing 0.5 mmol/l probenecid to prevent dye-leakage from the cytoplasmic space into the extracellular medium (22). The Ca^2+^-sensitive dye Rhod-2-AM (200–250 μl stock solution, 1 mg/ml in DMSO; AAT Bioquest, Sunnyvale) was added over a 5-min period, and the dye-containing solution (∼70–100 ml) was recirculated for 40 min. After completion of dye loading, hearts were perfused with bicarbonate-buffered solution to wash out any excess voltage- and Ca^2+^-sensitive dyes.

#### Guinea pig.

Langendorff-perfused guinea pig hearts were loaded with 22 μl of a solution containing the voltage-sensitive dye di-4-ANBDQBS (20 μl 29 mmol/l in ethanol; University of Connecticut Health Center) and Pluronic F-127 (2 μl of a 20% stock solution in DMSO; Life Technologies, Paisley, UK) through bolus injection over 4–5 min (at a Langendorff perfusion rate of 8–10 ml/min, the dye was diluted in 40–50 ml bicarbonate-buffered solution during application). The Ca^2+^-sensitive dye Cal-520-AM (200 μl, 1 mg/ml in DMSO; AAT Bioquest) was loaded after the voltage dye via bolus-injection and recirculated for 40 min as described above. Cal-520-AM has a spectrum very similar to Fluo-4, with an improved signal-to-noise ratio (48).

### Tissue Slice Preparation

It is essential to keep tissue immobilized during vibratome cutting. This reduces tissue damage, caused by movement relative to the cutting plane. In previous studies, 2,3-butanedione 2-monoxime (BDM) (31) was applied as electromechanical uncoupler (9–11), while blebbistatin is currently more widely accepted (65) for optical mapping (25). Both uncouplers were tested. After dye loading, hearts were perfused at room temperature with either BDM-containing HEPES-buffered solution (in mmol/l: 140 NaCl, 1.8 CaCl_2_, 5.4 KCl, 1 MgCl_2_, 11 glucose, 5 HEPES, 10 BDM, and 0.5 probenecid for Rhod-2-AM loaded hearts; bubbled with 99.9% medical grade O_2_; pH 7.4) or blebbistatin-containing (10 μmol/l) bicarbonate-buffered solution (contents described as above, with 0.5 mmol/l probenecid for Rhod-2-AM-loaded hearts), until the heart did not show any contractions.

The left ventricular (LV) free wall was removed from the heart, and the apex was cut off at about one-eighth of the total length of the heart. A cut was made counter clockwise from the apical end along the LV-septum border. This cut was continued below the circumflex artery along the coronary sulcus and then turned towards the apex after covering about two-thirds of the LV free wall width. Edges and any papillary muscles present were trimmed to enable flattening of the excised tissue before gluing it endocardium-down (histoacryl tissue adhesive; Braun, Melsungen, Germany) onto a block of 4% agar (low melting-temperature agar; Nusieve GTG agarose; Lonza), which in turn had been fixed on top of the vibratome cutting stage. The tissue block was cut in the epicardium-tangential plane, using a high precision vibratome (7000smz tissue slicer; Campden Instruments, Loughborough, UK) with a ceramic blade (Campden Instruments) at a progression speed of 0.03 mm/s (lateral blade vibration amplitude 2 mm, frequency 80 Hz). Slices were cut at a thickness of 350–400 μm. This was chosen to avoid hypoxic conditions in the tissue core, while preserving a good source of fluorescent signals. During the slicing procedure, the tissue was kept in ice-cold oxygenated BDM-containing HEPES-buffered solution (bubbled with 99.9% medical grade O_2_; pH 7.4 at 4°C) or blebbistatin-containing (10 μmol/l) bicarbonate-buffered solution (bubbled with 95% O_2_-5% CO_2_; pH titrated to 7.4 at 4°C).

Tissue slices were collected on thin blocks of Polydimethylsiloxane (Sylgard 184; Dow-Corning, Midland, MI) and held in position using a plastic-framed soft mesh. These assemblies were incubated in blebbistatin-containing bicarbonate-buffered solution for tissue recovery at 35 ± 2°C. For the assessment of the minimum recovery time needed to reach steady-state AP properties, measurements were taken at several time points after sectioning (from 5 min up to 3 h).

In a separate set of experiments, designed to explore the influence on AP properties of incubation temperature, rabbit cardiac tissue slices that had reached AP steady state after the initial incubation in warm (35 ± 2°C) bicarbonate-buffered solution were reexposed to ice-cold BDM-containing HEPES buffer for ∼1 h before moving them back to warm (35 ± 2°C) blebbistatin-containing bicarbonate-buffered solution for repeat measurements. *V*_m_ and CaT signals were recorded at multiple time points before and after reexposure to cold.

### Dual V_m_ and CaT Mapping of Tissue Slices

For imaging, tissue slices were kept in blebbistatin-containing bicarbonate-buffered solution at 35 ± 2°C. A LED (LED-CBT-90-R; peak wavelength: 624 nm; Luminus Devices, Billerica, MA) with excitation-filter D640/20× was used for excitation of the *V*_m_-sensitive dye. A white light illuminating LED (LED-CBT-90-W; Luminus Devices) was used for excitation of the Ca^2+^-sensitive dye, using excitation-filter S555/25× for Rhod-2-AM (rabbit) or D470/40× for Cal-520-AM (guinea pig). All filters were obtained from Chroma Technology (Bellows Falls, VT) and LED light collimated with a plano-convex lens (LA1951; Thorlabs, Ely, UK).

The *V*_m_ and CaT signals were collected by an EMCCD camera (Cascade 128+; Photometrics, Tucson, AZ). For rabbit tissue slice mapping (di-4-ANBDQPQ and Rhod-2-AM), a custom-made multiband filter ET585/50-800/200 (Chroma Technology) and a long-pass filter with significant transmission at wavelengths >575 nm (BLP01-561R-25; Semrock, Rochester, NY) were placed in front the camera lens (Navitar, Rochester, NY) for collection of fluorescence emission. For guinea pig slices (di-4-ANBDQBS and Cal-520-AM), a different custom-made multiband filter was used (ET525/50-800/200; Chroma Technology). The dual *V*_m_ and CaT imaging setup relied on the excitation isosbestic points for the voltage responses of di-4-ANBDQBS and di-4-ANBDQPQ, respectively (44, 46).

The EMCCD camera was used at its maximum resolution (128 × 128 pixels) and at a frame rate of 510 Hz. *V*_m_ and CaT data were obtained pseudosimultaneously using one camera, and *V*_m_ or Ca^2+^ dye excitation LED was switched on in a nonoverlapping frame-accurate 1:1 sequence (43). Each parameter was sampled with a frequency of 255 Hz. Linear interpolation was used to estimate data values between two consecutive measurements for each parameter to enable comparison of temporal correlation.

### Pacing

Four point electrodes (Lohmann Research, Castrop-Rauxel, Germany) and one set of parallel platinum electrodes were coupled to a tailor-made electrical stimulator for local and field stimulation, respectively. The four point electrodes were gently placed onto different locations of the tissue slices, as far from one another as possible. The two platinum electrodes (20 mm × 2 mm × 0.2 mm, placed ∼3.5 cm apart from each other) were aligned in parallel to the apex-base direction of the slice. We recorded *V*_m_ and CaT signals at 1-, 2-, 3-, 4-, and 5-Hz pacing; 15 APs for each frequency were recorded for each point stimulation site and for field stimulation. The stimulus amplitude was chosen to be 1.5 times the threshold voltage; pulse duration was 2 ms, using a bipolar stimulus to avoid deleterious electrochemical effects.

### Whole Heart Epicardial Imaging

Optical mapping on hearts (rabbit: *n* = 2; guinea pig *n* = 2) was performed before and in-between (below) slice collection to assess the dye loading and to collect functional parameters from subepicardial regions in the Langendorff-perfused intact heart. During whole heart imaging, the heart was perfused with blebbistatin-containing bicarbonate-buffered solution, with the LV free-wall facing the camera. Light source, excitation, and emission filters were the same as described above for tissue slice imaging in the two species, respectively.

### Collecting Subepicardial Slices from Langendorff-Perfused Heart

Guinea pig hearts were isolated, dye-loaded, perfused with bicarbonate buffer solution, and placed into the modified slicing chamber of a vibratome (Campden7000smz). The chamber was filled with solution (35 ± 2°C), and the epicardial *V*_m_ was optically mapped. Cardiac motion was restricted by perfusion with 10 μmol/l blebbistatin, while the heart was fixed gently in a silicone cradle. A cannula (gauge 16) was placed through the LV wall to avoid intraventricular fluid build-up. Tissue slices (∼400 μm) were cut tangentially to the mapped epicardial surface. The exposed surface was optically remapped, before each new cut. This process was repeated until the LV wall was completely sectioned. Slices were mounted as described earlier and transferred to an imaging chamber (35 ± 2°C) for optical mapping. Field stimulation was used for whole heart and tissue slice imaging.

### Assessment of Viability and Structure of Cardiac Slices

To assess the ultrastructural integrity of cardiac tissue slices, both transmission electron microscopy (TEM) and scanning electron microscopy (SEM) were used. Tissue was fixed with 2.5% buffered glutaraldehyde (Agar Scientific, Stansted, UK), postfixed with OsO_4_, dehydrated in acetone, and embedded for TEM in Epon. Samples for SEM were prepared in a similar manner, except they were critically point dried and sputter coated with gold. TEM samples were thin sectioned in the plane of the slice and examined on a JEOL 1200EX (Welwyn Garden City, UK). SEM samples were examined on a JEOL 35 SEM at 15 kV.

### Data Analysis

**Fig. 1.**
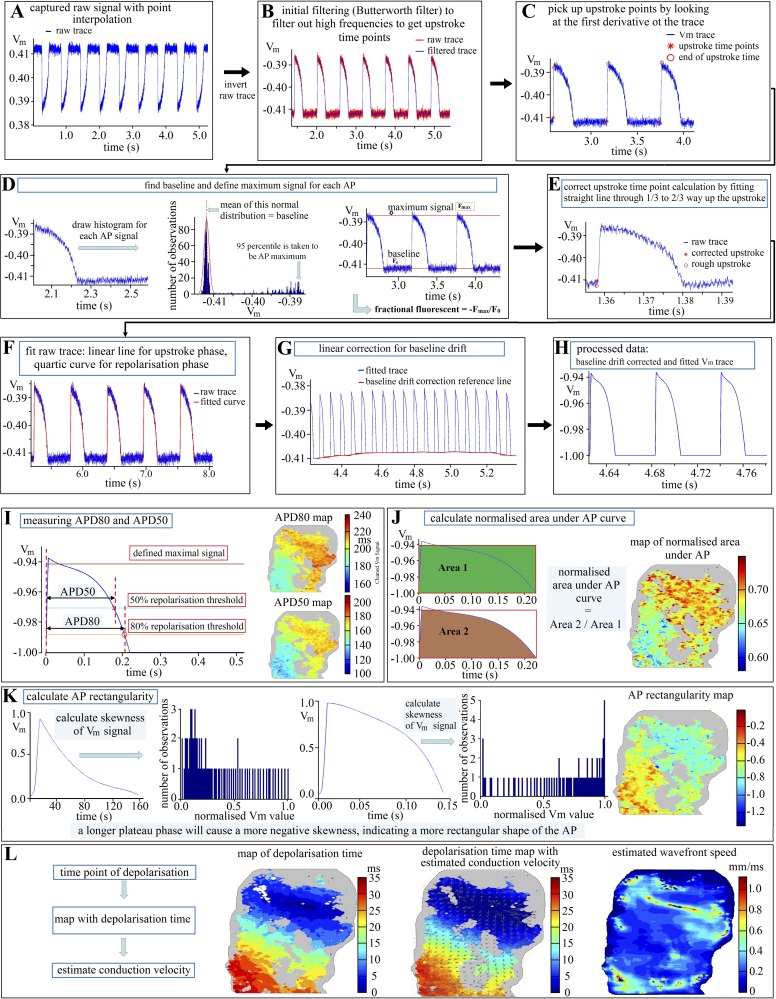
Flow chart of data processing and parameter extraction: *A*: raw transmembrane potential (*V*_m_) signal trace; *B*: low-pass frequency filtering with a Butterworth filter (stop band: 75 Hz; pass band: 60 Hz) to estimate upstroke time point; *C*: preliminary identification of depolarization (or upstroke) timing from the filtered trace; *D*: identification of action potential (AP) maximum and *V*_m_ baseline from the raw signal trace and calculation of 95th percentile and definition as maximal AP amplitude for further processing; *E*: correction for the identified upstroke time points; *F*: polynomial fitting through raw data points; *G*: linear correction for baseline drift; *H*: processed *V*_m_ trace; *I*: estimating action potential duration at 50% and 80% repolarization (APD_50_ and APD_80_), based on defined maximal signal (as shown in *D*); *J*: calculating area under AP curve and the AP area ratio; *K*: calculating AP skewness; *L*: calculated conduction velocity. Grey regions in *I*–*L* indicate tissue with weak or no signal [(F_max_ − F_0_)/F_0_ × 100% <0.3%]. See *Data Analysis* for detail.

Due to the large amount of data collected in each experiment, a semiautomatic data analysis tool was written in Matlab (The MathWorks, Natick, MA), which analyzes data sets to extract relevant information. The details for *V*_m_ signal processing and parameter estimation are shown in Fig. 1 as a flowchart; CaT signal processing was conducted in a similar manner.

**Fig. 2.**
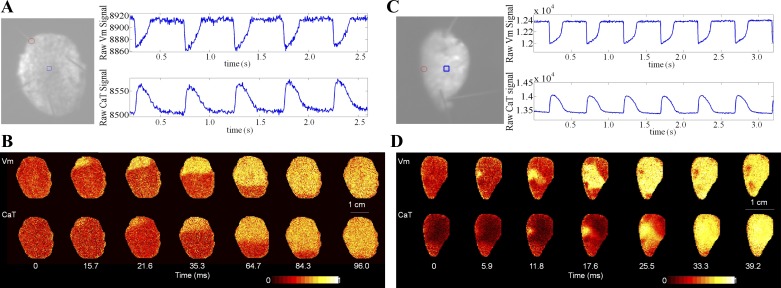
Raw signal from dual *V*_m_ and calcium transient (CaT) mapping of a left ventricular tissue slice of rabbit (*A* and *B*) and guinea pig (*C* and *D*). *A*, *left*: image of rabbit tissue slice, with red circle indicating the stimulation point and blue square indicating the area (3 × 3 pixels) used to plot *V*_m_ and CaT traces (di-4-ANBDQPQ and Rhod-2-AM; 2 Hz pacing). *B*: normalized fluorescence intensity maps for *V*_m_ and CaT from the sample in *A* at 7 time points, showing the progression of V_m_ and CaT activation waves. *C*, *left*: image of guinea pig tissue slice with red circle indicating the stimulation site and the blue square showing the region (3 × 3 pixels) used for raw *V*_m_ and CaT traces (di-4-ANBDQBS with Cal-520-AM; 2 Hz pacing). *D*: normalized fluorescence intensity maps of V_m_ and CaT from the sample in *C* at 7 time points, showing the progression of *V*_m_ and CaT activation waves.

**Fig. 3.**
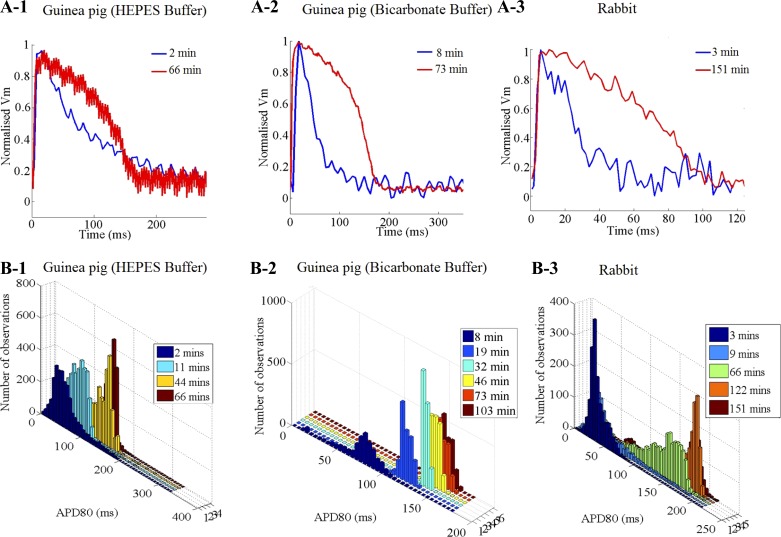
Recovery in AP properties after cutting of tissue slices in ice-cold solution. *A*: unfiltered *V*_m_ traces, averaged over the whole slice, for a guinea pig left ventricular slice at 2 min (blue) and 66 min (red) after removal from ice-cold BDM containing HEPES buffer (*A-1*); a guinea pig left ventricular slice, 8 min (blue) and 73 min (red) after removal from ice-cold blebbistatin containing bicarbonate buffer (*A-2*); and a rabbit left ventricular slice, 3 min (blue) and 151 min (red) after removal from ice-cold BDM containing HEPES buffer (*A-3*). *B-1*, *B-2*, and *B-3*: APD_80_ histograms of the slices above at several postcutting time points during a 2-Hz field stimulation. Time after cutting is color coded.

**Fig. 4.**
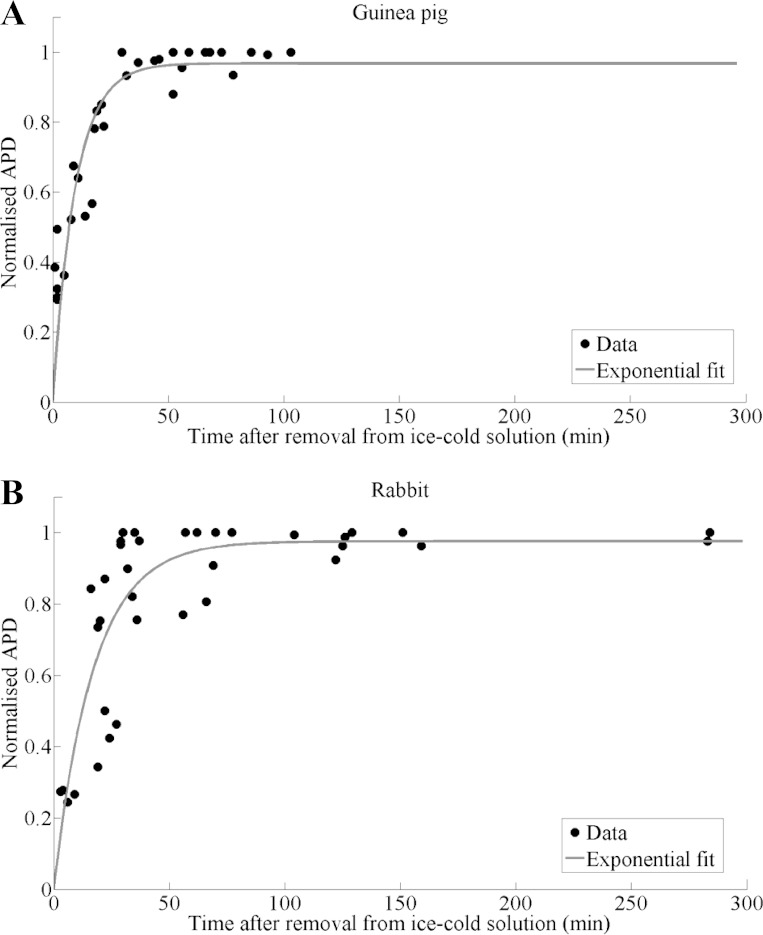
Change in APD after cutting in ice-cold solution: Summary of normalized mean APD_80_ during 2-Hz pacing at different time points after cutting for guinea pig and rabbit slices (*A*: 8 slices from 5 guinea pigs; *B*: 7 slices from 4 rabbits; solid curves show exponential fit).

Signal processing was necessary to reduce the impact of noise upon parameter estimation (e.g., APD was calculated from processed *V*_m_ signal traces). Recorded signal traces were processed through the following steps: *1*) identifying the depolarization timing on the filtered trace to break up the recorded signal trace into individual AP cycles (Fig. 1, *A*–*C*); *2*) estimating the maximum (F_max_) and baseline (F_0_) fluorescence intensities for each identified AP, and calculating the corresponding fractional fluorescence [“(Fmax − F0)/F0” × 100, to give the percentage of the fluorescence ratio; Fig. 1*D*]; *3*) correcting the initially identified depolarization time point if needed (Fig. 1*E*); *4*) polynomial fitting (1st order for depolarization phase, 4th order for repolarization phase) through raw data points (Fig. 1*F*); and *5*) linear correction of baseline drift (Fig. 1*G*). Very weak signals (i.e., where the fractional fluorescence was <0.3%) were classified as “no signal.” In the Online Supplement an example is shown of raw and processed *V*_m_ data from a representative tissue slice (from rabbit; see Supplemental Movie “DataProcessingExample”; Supplemental Material for this article is available online at the Journal website): both de- and repolarization characteristics can be identified more robustly from the processed data.

Particular care needs to be taken when determining maximum AP amplitudes, as this feeds through to the calculation of APD, customarily defined at set percent repolarization levels (relative to maximal depolarization). Maximum AP amplitude is defined by a single data point, which makes peak values extremely sensitive to noise and sampling rate in experimental measurements. This can give rise to artificially exaggerated differences in AP amplitudes, with consequent spurious changes in APD estimation, even when underlying AP shapes are very similar. To obtain more physiologically relevant and robust APD data, both for experimental measurements and simulated traces, a more reproducible identification of the AP “peak value” is needed. Here, for each AP signal, we use the 95th percentile of the signal (i.e., the value below which 95% of all data points of the AP signal fall) as a surrogate for the (apparent) absolute peak value. Figure 1*I* shows one AP from an optical mapping measurement (preprocessed with procedure described before) with defined maximal value, APD at 50% repolarization (APD_50_) and APD at 80% repolarization (APD_80_) marked out. For our tissue slice optical mapping data, APD_80_ was used instead of APD at 90% repolarization (APD_90_) to reduce the impact of baseline noise on APD estimation. After APDs were calculated for each pixel, APD maps were generated (averaged APD over a number of AP cycles). An example of APD_80_ and APD_50_ maps is shown in Fig. 1*I* (note: grey color coding is used to identify areas within the physical dimensions of a slice, where insufficient fractional fluorescence was observed; classified as “no signal,” see above). The threshold for “no signal” classification can be adjusted, according to experimental needs. Fluorescence signal strength depends on dye loading, tissue integrity/viability, and underlying structures (e.g., cleavage planes between cell layers). It is common to have uneven signal strengths in cardiac tissue, including slices, and the size of “no signal” areas varies.

To characterize AP shape quantitatively in an automated manner, two indicators were used. The first is the area under AP curve, normalized to the rectangle defined by AP amplitude (95th percentile) and APD (Fig. 1*J*, henceforth called the “AP area ratio”). The smaller the value of this ratio, the less rectangular is the shape of the AP. The second indicator is referred to as the “AP skewness,” and this is calculated as the third standardized moment of the distribution of all points constituting the processed AP signal trace. The more right-shifted the peak of the *V*_m_ distribution during the AP (generating a tail of the distribution predominantly on the *left*), the more negative is the AP skewness. In Fig. 1*K*, two AP examples are given to demonstrate the use of AP shape indicators. The *left-hand side* of Fig. 1*K* shows an AP with triangular shape. This would yield in a positive skewness with a small value in AP area ratio. An AP with a pronounced plateau phase is shown at the *right-hand side*, and this AP has a negative skewness and a bigger area ratio.

Together, AP area ratio and AP skewness provide user-independent and quantitative characterizations of AP shape (in terms of parameters such as rectangularity or plateau) allowing for reliable and automated identification of changes in AP shape after an intervention and comparison of AP shapes between individual samples. From the regionally resolved upstroke timing in the processed data, conduction velocity was estimated using Bayly's method (5), and the activation wavefront speed was calculated from velocity vectors identified based on activation timing across the slice (see Fig. 1*L*).

Before each signal trace was processed, spatial filtering (such as mean or Gaussian) can be applied to the optical mapping images. In our case, a mean filter with a range of three pixels was applied (i.e., the averaged value of a 3 × 3 pixel square was taken as the new value of the central pixel in this square). After spatial filtering, the signal trace (i.e., fluorescence intensity change over time) was read out for each pixel (in the case of dual *V*_m_ and CaT mapping, *V*_m_, and CaT traces were separated) and processed with the methods shown in Fig. 1, *A*–*H*. Functional parameters (e.g., APD and AP shape indicators) were calculated for each processed AP (or CaT) from the signal trace (as shown in Fig. 1, *I*–*L*). These functional parameters were then grouped by pacing frequencies, and for each pacing frequency the average functional parameter value was identified (for example, 15 steady-state APs were recorded at 2-Hz pacing, and the representative APD value was the average of the 15 APDs). Functional parameter maps (like the APD_80_ maps shown in Fig. 1*I* and the AP skewness map shown in Fig. 1*K*) were plotted for each pacing frequency, with each point on the map representing the averaged functional parameter value per slice.

### Statistical Analysis

To quantify the postcutting recovery time, an exponential curve:

$${\text{ADP}}_{80\text{normalised}}=\text{A}\left(1-e^{-\text{Bt}}\right)$$

was fitted through the normalized APD_80_ data (mean APD_80_ for the whole slice), obtained at several postcutting time points (APD_80_ at each postcutting time point is normalized to the maximal APD_80_ value observed over the entire time interval). In the formula, *A* and *B* are constants to be fitted and *t* is the time (in min) after removing the slice from ice-cold solution. The fitting was performed using Matlab curve fitting tools.

To compare the recovery dynamics of the two species (i.e., rabbit and guinea pig) and to assess any difference in the recovery dynamics caused by the use of different solutions (i.e., BDM-containing HEPES buffer, blebbistatin-containing bicarbonate buffer), both *F*-tests and *t*-tests were performed.

For the *F*-test (55), the null hypothesis is that one curve can fit through all data points (2 species, 2 solutions) and that this fit is as good as fitting two groups of data points separately. The formula for *F*-ratio calculation can be found in Ref. 55.

The *t*-test is performed to assess whether two groups of data have two significantly different recovery times (time to reach steady-state AP). We concentrate on the assessment of the fitted constant *B* (without extra assessment of *A*), since the recovery time is dependent only on *B*. For example, the time needed to reach 97.5% of the final AP steady-state equals $\frac{\ln\left(0.025\right)}{-B}$. The *t* value is calculated (in a nonstandard manner) as:

t=∣B^1−B^2∣SE12+SE22

where ${\hat{B}}_{1}$ and ${\hat{B}}_{2}$ are the best-fit of constant *B* for groups one and two, respectively, and SE_1_ and SE_2_ are the standard error of the fitted constant *B* for groups one and two, respectively (this can be readout from Matlab regression tool). The total number of degrees of freedom is the sum of degrees of freedom of the two groups. After the *t* value is calculated, the *P* value can be obtained.

## RESULTS

### Dual V_m_ and CaT Measurements

Two dye combinations were used for dual *V*_m_ and CaT mapping of tissue slices: di-4-ANBDQPQ in combination with Rhod-2-AM for rabbit, and di-4-ANBDQBS with Cal-520-AM for guinea pig heart. Figure 2 shows the raw signals, acquired from one rabbit (Fig. 2*A*) and one guinea pig (Fig. 2*C*) LV tissue slice. Figure 2, *B* and *D*, shows 2D maps of the progression of *V*_m_ (*top*) and CaT activation waves (*bottom*) after point stimulation at locations identified in Fig. 2*A*. The delay in onset of CaT activation, compared with start of AP upstroke, was 2.21 ± 0.60 ms in rabbit (mean ± SD; 2 animals, 5 slices) and 2.33 ± 1.00 ms in guinea pig (means ± SD; 2 animals, 9 slices). No significant difference in AP to CaT activation was identified between the two species (*P* = 0.81).

Pacing frequency-dependent responses of APD and CaT duration, as described before (60), were observed in both species (shortening of both APD and CaT at higher pacing rates; data not shown).

### Time to Reach Equilibrium After Slicing

In both species, sharp, short, and triangular APs without a clear plateau phase were observed immediately after cutting the tissue in ice-cold solution, both for BDM-containing HEPES buffer and blebbistatin-containing bicarbonate buffer. Figure 3, *A-1* and *A-2* (guinea pig) and *A-3* (rabbit), showed unfiltered (unprocessed), normalized *V*_m_ signals (averaged over the whole slice), recorded immediately after cutting, and subsequent to incubation at body temperature for over 1 h. Figure 3, *B-1*, *B-2*, and *B-3*, shows APD_80_ histograms (2-Hz pacing) of the same guinea pig and rabbit slices as shown in Fig. 3, *A-1*, *A-2*, and *A-3*, measured at several postcutting time points. An increase in APD_80_ was observed after cutting, until a steady state was reached. In Table 1, AP descriptors (AP area ratio and skewness) of a guinea pig slice (AP signal in Fig. 3*A-1*) measured at two postcutting time points (2 and 66 min), together with the AP descriptors obtained from the same heart (LV epicardial view) before cutting. Compared with the AP obtained shortly after cutting, APs recorded after a 1-h incubation had a shape much closer to that measured in the whole heart before sectioning.

**Table 1.** Example of AP shape indicators, obtained from a guinea pig tissue slice before and after cutting

	Average AP Area Ratio	Average AP Skewness
Guinea pig whole heart: before cutting (epicardial aspect of LV)	0.751 ± 0.08	−1.157 ± 0.061
Guinea pig tissue slice: 2 min after cutting	0.567 ± 0.076	0.010 ± 0.243
Guinea pig tissue slice: 66 min after cutting	0.718 ± 0.012	−0.960 ± 0.075

Numbers (*n*) used to calculate means ± SD values are the number of pixels capturing transmembrane potential (*V*_m_) signal from the guinea pig tissue slice or the guinea pig whole heart epicardial surface; *n* number equals to 3,547 for the slice and 8,428 for the whole heart. AP, action potential; LV, left ventricle.

For quantification of the postcutting recovery time needed to reach steady-state AP properties, an exponential curve was fitted through the normalized APD_80_ values, measured at different postcutting time points. The time to reach 97.5% of the final steady-state APD_80_ values was calculated.

To assess whether transient exposure to BDM and/or HEPES buffer (guinea pig, 3 slices, 2 animals) during slice preparation was a major contributor to the AP shortening and triangular shape seen after cutting, the process was repeated in ice-cold bicarbonate-buffered solution containing blebbistatin (guinea pig, 5 slices, 2 animals). Note that all slices, whether cut in BDM or blebbistatin-containing solution, were rewarmed in the same buffer (blebbistatin-containing bicarbonate buffer) immediately after cutting, to focus on cutting conditions. Neither *F*- nor *t*-test identified any differences in AP recovery dynamics or time after the slicing procedure (*P* = 0.725 for *F*-test; *P* = 0.096 for *t*-test).

Changes in normalized APD_80_ (mean APD_80_ over the whole slice) were measured at several postcutting time points from guinea pig (8 slices, 4 animals) and rabbit (7 slices, 4 animals) slices, respectively, during a 2-Hz field stimulation (Fig. 4, *A* and *B*) The fitted exponential curves are shown as solid lines. The time to reach 97.5% of steady-state APD_80_ values was 36 and 63 min for guinea pig and rabbit, respectively. Both *F*-test and *t*-test identified a significant difference between recovery dynamics of the two species at 5% significance level (*P* = 0.006 and *P* = 0.008, respectively).

**Fig. 5.**
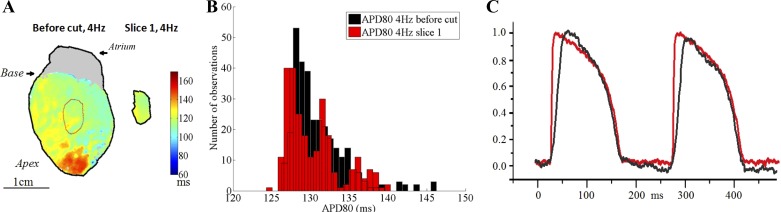
Comparison of AP properties in matching tissue optically mapped in whole Langendorff-perfused guinea pig heart and in a tissue slices cut subsequently at body temperature (blebbistatin-containing bicarbonate buffer). *A*: subepicardial APD_80_ map of the whole perfused left ventricle (LV), with the subsequently sectioned area outlined in red, and from the corresponding subepicardial tissue slice (*slice 1*) during field stimulation at 4 Hz. *B*: histogram showing APD_8_0 values for the LV area circled in red (in *A*) and from the corresponding subepicardial tissue slice, during field stimulation at 4 Hz. *C*: averaged *V*_m_ traces from the perfused whole heart LV (black) and the subepicardial tissue slice (red).

In slices obtained by sectioning guinea pig Langendorff-perfused heart at 37°C, we did not observe sharp and short APs shortly after cutting (Fig. 5). AP shapes closely resembled those collected from the tissue before cutting. Figure 5*A* shows the APD_80_ maps from the LV epicardium/subepicardium of a Langendorff-perfused heart and from the subsequently taken subepicardial tissue slice, here at a 4-Hz field stimulation. In Fig. 5*A*, the red outline on top of the whole heart APD_80_ map indicated the area where the next slice was taken. The APD_80_ histograms of the area inside the red outline in the perfused whole heart and from the subsequently taken tissue slice, here mapped 8 min after cutting, are shown in Fig. 5*B*. In Fig. 5*C*, averaged AP traces are shown from the subepicardial region inside the red outline of the perfused heart before cutting (black) and of the subsequently taken tissue slice (red). The slices and the whole heart were paced by field stimulation. In the whole heart, field simulation was not sufficient to activate the whole heart at the same time; there was still a gradient of activation time through the ventricular wall. Although a blue light source was used to reduce light penetration into the tissue, signals from deeper myocardial layers were also collected, which will explain why the AP upstroke appears slower in the whole heart than in the pseudo-2D slice.

**Fig. 6.**
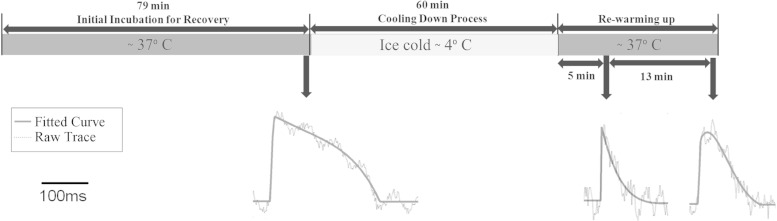
Influence of ice-cold temperature on AP properties: spatially averaged AP traces (averaged over the whole slice) measured at body temperature (79 min after initial cutting) and again at 5 and 18 min after a 1-h reexposure to ice-cold solution.

In Fig. 6, we show spatially averaged AP traces (2-Hz field stimulation) of a rabbit slice at steady state after the initial sectioning (79 min postcutting) and at 5 min and 18 min after reexposure to ice-cold solution (for 60 min); all recordings at body temperature. Shortly (5 min) after the rewarming process was started, a short and sharp AP is observed, which shows a trend towards increasing APD over time (18 min). This illustrates that exposure to low temperature appears to play a critical role in causing the observed changes in AP shape and duration.

### Multiple Point Stimulation

Previous cardiac tissue slice studies tended to use one site for AP stimulation (10, 11, 57). To explore whether pacing location affects functional parameters (e.g., APD_80_), we used four different point stimulation sites on each of the slices and field stimulation as a reference case. APD_80_ maps obtained with these protocols were compared. To exclude effects from the initial changes associated with tissue recovery (above), data for these comparisons were obtained after at least 70 min storage at body temperature. Measurements were repeated over the course of an hour, with each set of observations consisting of five measurements (4 point and 1 field stimulation) taken in swift succession.

**Fig. 7.**
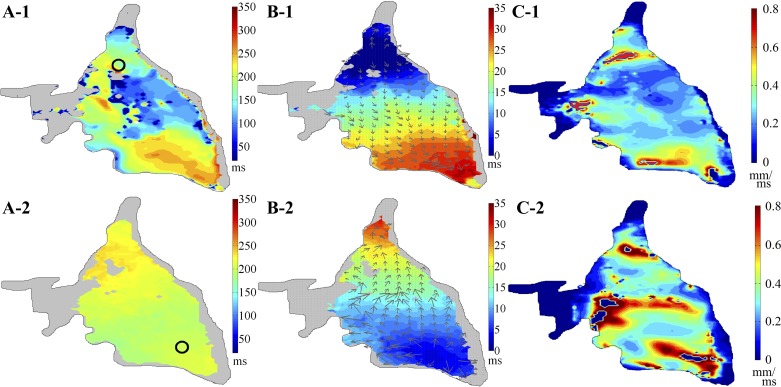
APD_80_, conduction velocity, and conduction speed maps of a rabbit slice, paced from 2 different locations (here at 1 Hz). *A-1* and *A-2*: APD_80_ maps, with pacing sites indicated by black circles. *B-1* and *B-2*: conduction velocity (grey arrows) plotted on top of depolarization time map. *C-1* and *C-2*: conduction speed (in mm/ms). Grey areas indicate tissue with no significant florescent signal amplitude.

The pacing-site dependent differences in APD were observed in most slices, at all the pacing frequencies tested. Differences could be pronounced, as shown in Fig. 7, where pacing from one end of the slice yielded a region of very short APD that was absent upon pacing from the other end (compare Fig. 7, *A-1* and *A-2*). Conduction velocity (Fig. 7*B-1*, vectors plotted on top of the depolarization time map, where the size of the vector is proportional to the magnitude of the conduction velocity) and conduction speed maps for the first pacing location (Fig. 7*C-1*) show that slowed conduction coexists with the short APD. When pacing from the other end, both conduction velocity and speed (Fig. 7, *B-2* and *C-2*) were more inhomogeneous, matching the APD distribution.

### Assessment of Tissue Slice Viability

**Fig. 8.**
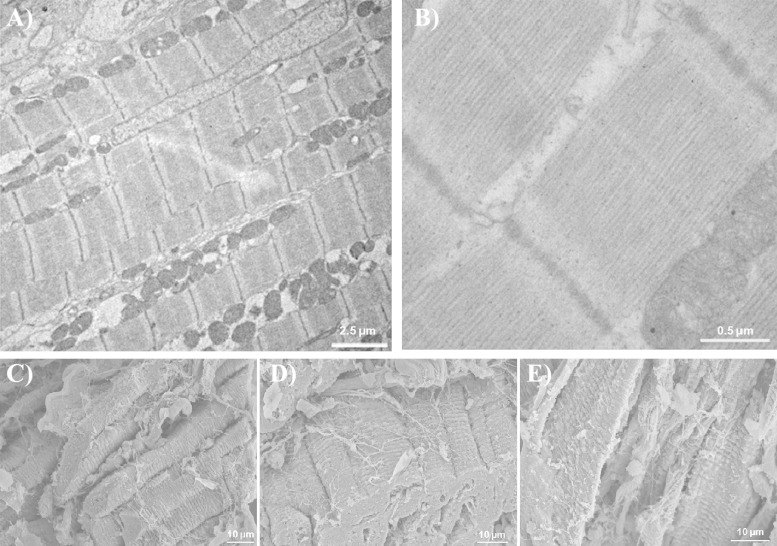
Transmission electron microscopy (TEM) and scanning electron microscopy (SEM) images of rabbit slice tissue, fixed at different time points after cutting. *A*: TEM images 5 min after cutting, overview of intact cellular membrane structures (sarcolemma, mitochondria, T tubules) and regular cross-striation (z-lines). *B*: enlarged view of sarcomeric protein structures with well-aligned isotropic and anisotropic bands. Magnification: ×7,500 in *A* and ×50,000 in *B*. *C*, *D*, and *E*: SEM images of tissue samples taken at after 5 min, 1 h, and 4 h; respectively. All slices show cells with intact sarcolemma, preserved extracellular structures, and regular sarcomeric striation patterns.

To assess ultrastructural integrity of tissue slices, SEM and TEM were conducted on samples from tissue slices at ∼20 μm below the sectioned surface. The TEM in Fig. 8 illustrates that subcellular structures, including well-aligned contractile fibers, intact mitochondria, and nuclei during a 4-h period after cutting, were normal in appearance. Spatial relationships of myocytes, extracellular collagen, fibroblasts, and vasculature all appeared normal by SEM.

## DISCUSSION

### Dual V_m_ and CaT Optical Mapping

The main advantages of optical mapping are high spatial resolution, capacity to monitor multiple physiological parameters, and the ability to do so simultaneously. In addition, voltage-sensitive dyes can record AP shapes rather than activation and repolarization timings only.

Dual *V*_m_ and CaT mapping has been used frequently for whole heart studies (15, 17, 18, 50) but has had limited application to cardiac tissue slices (45). *V*_m_ and CaT are key parameters of cardiac function, and their relationship is crucial for electromechanical coupling and mechanoelectric feedback in the heart (52). Combined *V*_m_ and CaT optical mapping allows one to study not only the individual characteristics of these two parameters (e.g., the frequency response of *V*_m_ and CaT) but also their spatiotemporal interrelation (16). Monitoring both parameters simultaneously can be beneficial for testing pharmacological interventions, which may affect *V*_m_ and CaT independently. Finally, dynamic high-resolution observation allows one to identify conduction wavefront maps and spatial distribution of key parameters (e.g., APD), which is crucial, for example, for studying ventricular fibrillation (4, 14).

We used two dye-combinations for combined *V*_m_ and CaT mapping and demonstrated their utility in two species (Fig. 2). These combinations were not species-specific but application to other species could require modification of excitation and emission filters since the excitation isosbestic points of di-4-ANBDQPQ and di-4-ANBDQBS vary [e.g., the excitation isosbestic point of rabbit is at a longer wavelength compared with rat (46)].

Both di-4-ANBDQPQ and di-4-ANBDQBS are voltage-sensitive dyes of an increasingly wide range of dyes with different spectral characteristics (73) that combine significantly improved *V*_m_ signal with internalization stability (53). These characteristics make them ideal for tissue slice optical mapping, as the thin tissue preparations yield less signal compared with arterially perfused large tissue blocks.

Both Rhod-2-AM and Cal-520-AM have relatively high calcium affinities (*K*_d_ ∼570 and∼320 nM, respectively; data sheets from AAT Bioquest). High-affinity dyes bind calcium during early systole. Slow release of calcium from these dyes may generate inaccuracies in calcium amplitude and apparent calcium decay dynamics (40). Figure 2 shows that both CaT traces display a fast and slow decay phase; the slow decay phase may, in part at least, be a consequence of relatively slow calcium unbinding from the dye. Further investigations of CaT properties, including comparisons of CaT signals recorded with high- and low-affinity calcium dyes, would be necessary (40).

### Cardiac Tissue Slices

Although cardiac slices are not as popular yet as isolated cell or Langendorff heart preparations, their moderate complexity can offer unique insights into cardiac electrophysiology. Considering the complexity of the system and its ability to capture in vivo-like phenomena, tissue slices can bridge the gap between those two models. Compared with single cells, slices offer preserved cell-cell connections that enable one to study tissue-level phenomena (e.g., conduction), while the locally preserved extracellular microenvironment avoids potential pitfalls of changes in functional properties due to the loss of extracellular matrix or damage by the cell isolation process. Compared with whole heart, tissue slices represent a simpler model, which makes the interpretation of structure-function relationships (e.g., the correlation between cell direction and conduction speed) easier. Tissue slices also allow full access to midmyocardial tissue, which is difficult in the perfused whole heart. They can be cultured (32) and used over several days, which opens up the possibility of maintaining biopsy material, for example from patients, for long-term investigation (6, 11).

### Postslicing Recovery Time

There is a critical recovery time-period, required for cardiac slices to reach an electrophysiological steady-state. During our recovery protocol, we observed, both in rabbit and guinea pig slices, short and triangular APs right after cutting (e.g., Fig. 3). In addition to the short and sharp triangular APs, tissue slices showed relatively weak fractional fluorescence immediately after removal from the ice-cold solution used during cutting, followed by a rapid increase in signal strength. This occurred largely during the first half hour of the recovery period. To quantify the minimum recovery time in our setting, an exponential function was fitted through the normalized data from guinea pig and rabbit. The recovery times needed to reach 97.5% of steady-state APD_80_ values were 36 and 63 min for guinea pig and rabbit, respectively. These findings are consistent with previous reports (7), which highlighted that the success rate for electrophysiological measurements on cardiac tissue slices from neonatal rat, using patch clamp, increased after 30 to 60 min of incubation, supporting the frequently chosen slice recovery time of ∼1 h (20, 56). *F*- and *t*-tests suggest significant differences in the postcutting recovery dynamics and the recovery time between the two species. This highlights the need to verify the postcutting recovery time when applying this methodology to other species.

There are several possible explanations for the need to allow a recovery period before use of slices: *1*) BDM, used by many teams during cutting, may need to be washed out; *2*) some or all of the tissue may have become hypoxic during slicing; *3*) tissue slicing is associated with severe damage of cells near the cut surfaces, from which the tissue may need to recover; and *4*) during slicing, the tissue is kept in ice-cold solution, and the return to body temperature may affect its electrophysiological properties.

To assess the first possibility, we performed slicing in cold bicarbonate-buffered blebbistatin-containing (instead of HEPES-buffered BDM-containing) solution. Similar short and sharp AP configurations, and weak fluorescence signals, were observed immediately after cutting. The recovery dynamics of slices cut in these two different buffers did not show a significant difference. This suggests that the kind of uncoupler and the buffer system used are not an issue per se.

The second scenario, hypoxic damage, was countered in as far as that is possible by keeping the tissue in ice-cold oxygenated buffer solution during sectioning. This, together with electromechanical uncoupling, reduces metabolic demand. Interestingly, slices, taken from the well-oxygenated outer surface of the tissue block and those taken later and from deeper transmural planes showed no systematic difference in signal quality and recovery dynamics. Therefore, we conclude that it is unlikely that the observed sharp and short AP configurations arise in direct consequence of potentially hypoxic conditions in the tissue block.

To explore the third and the fourth possibilities (i.e., the tissue damage and the temperature effects), we sliced guinea pig whole hearts during Langendorff perfusion with warm (35 ± 2°C) blebbistatin-containing bicarbonate-buffered solution to obtain near-epicardial tissue slices. In this setting, no sharp and short APs were observed after slicing. Instead, APs, recorded from these slices shortly after sectioning, showed repolarization morphology and APD that was very similar to the presectioning intact tissue (Fig. 5). These results exclude tissue damage as the primary cause of the postcutting recovery phenomenon and suggest that the change in temperature is a probable main contributor.

Effects of temperature on AP properties have been observed before (24), generally reporting AP lengthening when lowering the temperature from body to room temperature (36, 51). Less is known about the effects on the AP of transient exposure of cardiac tissue to ice-cold solution. To further explore whether the ice-cold temperature the tissue experiences during slicing is the main reason for the sharp and short APs, observed shortly after cutting, fully recovered slices with normal AP configurations (*n* = 3) were reexposed to ice-cold BDM-containing HEPES-buffered solution for a period of 60 min and then mapped again. Indeed, the sharp and short AP shape, seen during the initial recovery phase, was observed again (Fig. 6). We conclude, therefore, that the use of ice-cold solution, necessary to avoid ischemic conditions in the tissue block during slice preparation, is a key contributor to the transiently abridged AP configurations and the observed electrophysiological recovery dynamics.

One possible explanation for these effects could be changes in cell excitability at a low temperature. Only part of the cells in a slice may be excited shortly after removal from ice-cold solution, with the portion of excitable cells increasing over time in warm solution. The nonexcited cells would then act as current sinks and therefore truncate the AP. The relatively weak signal shortly after sectioning is in keeping with this possibility, as discussed by others (30).

For slices collected from whole heart at body temperature, no postcutting recovery time was required to record near steady-state APs. However, slicing whole heart at body temperature has its limitations. As sectioning progresses, coronary arteries (which run initially at the epicardial/subepicardial surface) get cut, and perfusion of the heart is disturbed. At body temperature, where metabolic demand is relatively high, the heart may quickly enter ischemia even after cutting just one slice. Therefore, this approach is not suitable for studying multiple transmural slices.

### Frequency Response and Upstroke Time Delay Between AP and CaT

Shortening of APD with increasing pacing frequency is a well-known response of cardiac muscle (2, 26), and shortening of CaT duration has also been observed (38). Rate-dependent reductions in APD and CaT duration were seen in our LV tissue slices, both from guinea pig and rabbit.

The delay between onset of upstroke of AP and CaT has been measured to be just over 2 ms, both for guinea pig and rabbit (nonsignificant difference between species). Values in the same order of magnitude have been reported before (47, 49). Longer (17, 62) delays in CaT upstroke (compared to AP upstroke) have also been observed. One reason for this discrepancy is related to the method used to identify the different time points to calculate the CaT delay. These range from “earliest onset” of AP and CaT upstroke (62) to time points corresponding to the AP and CaT peak values (43). Differences may also stem from different measurement techniques [patch clamp (47) vs. optical mapping (49)], use of calcium-sensitive dyes with different sensitivity, or use of uncouplers (such as BDM) that affect the L-type calcium channel (1, 31, 37). Overall, the delays reported here are in agreement with values reported using patch clamp (47), optical mapping (49), and computational simulation (68).

### Multiple Point Stimulation

AP shape, APD distribution, and conduction dynamics at any given location in the slice can differ, depending on pacing site (Fig. 7). A probable explanation for this is the presence of nonuniform source-sink relations in the tissue, which has previously been shown to induce differences in conduction and APD in cell culture (76) and whole heart (64). In slices, there is the additional effect of variable cell alignment relative to the plane of the section. Although an epicardium-tangential cutting plane maximizes the proportion of myocytes that are aligned roughly in-plane, cells orientated nontangentially to the cutting plane are present, with increasing proportions as one moves away from the epicardium. One needs to be careful, therefore, when comparing conduction speeds from different slices, and it may be necessary to perform histological analysis to assess underlying cell orientations. In addition, cells within a slice come from different transmural depth, as the cut is conducted in a true plane, not one that emulates an “onion shell” of equidistant position relative to the ventricular surface (so cells in the centre of a slice will be from deeper transmural layers than those at the periphery). Thus, in spite of all efforts, slices will contain nonuniform structures, with varying degrees of cell alignment and coupling. These differences may create asymmetrical source-sink relations, which may reveal themselves, when pacing at different locations, in the form of differences in APD and conduction velocity.

Identification of an unbiased picture of APD distribution in tissue slices is therefore not possible with single point stimulation. Instead, multiple pacing points, and/or field stimulation, are called for. This is important for intervention testing, such as for drug application/washout studies, in particular if the pacing site is not controlled. If, for example, the pacing location varies before and after an intervention, changes in functional parameters that are due to source-sink heterogeneities could be mistakenly attributed to the effects of the intervention. Field stimulation would be more reliable for a before-and-after comparison, but the strength and orientation of the electric field relative to the tissue slice should also be controlled.

### Data Analysis and Hypotheses for Further Investigation

Multiparametric optical mapping generates large amounts of data per experiment, which makes data processing challenging. It is useful to employ automated or semiautomated routines, which analyze data sets, extract relevant information, save results, and generate maps and plots for user inspection. In this article, we describe our approach to data processing and parameter extraction to characterize AP and CaT. Some of the parameters are standard (e.g., conduction velocity). Two new parameters, normalized area under AP curve (the AP area ratio) and AP skewness, were added into the routine to better characterize the shape of AP. Both may be useful for other researchers who wish to quantify and compare AP shapes “automatically” without qualitative visual comparison.

We modified the method to evaluate APD by replacing the absolute upstroke peak value with the 95th percentile of the *V*_m_ signal amplitude. This reduces the overly sensitive dependence of APD on the AP peak values. The most suitable percentile value for this approach will depend on individual experimental conditions and AP shapes; in our analysis routine, this can be adjusted with no difficulty.

As fluorescence signals from tissue slices are weaker than those from perfused hearts or tissue wedges, it is important to process signal traces before parameter evaluation to increase the reliability of the information extracted. As illustrated in the Online Supplemental Movie, processing significantly reduces the impact of noise distortion on depolarization and repolarization phase detection. Multiple routines for processing optical mapping data have been published, including the one from Laughner et al. (42) for processing AP signals, mapped from whole hearts, with detailed explanation and comparison of spatial and temporary filters. Tian et al. (67) published their method to process single cell CaT signals, which is based on fitting a biexponential curve to the decay phase of the CaT trace. We used an alternative curve-fitting-based routine for processing AP signals. As shown in Fig. 1, first order (for depolarization) and fourth order polynomials (repolarization) were chosen for curve-fitting. This works reasonably well for signals obtained from tissue slices (which tend to be more “noisy” than signals from whole heart). For stronger signals with large signal-to-noise ratio, more computationally efficient temporal filtering (IIR or FIR filtering) can be sufficient.

Averaging over a series of APs, obtained at the same pacing conditions without additional interventions, is a good way to improve signal-to-noise ratio. This was not used in our routine to retain the ability for analyzing AP traces recorded during dynamic restitution protocols and to explore beat-to-beat variability. The maximum upstroke velocity of the AP (d*V*/d*t*_max_) is an important parameter; however, limits in temporal resolution (∼255 Hz for dual *V*_m_/CaT measurement, ∼510 Hz for single-parameter mapping) make estimation of d*V*/d*t*_max_ challenging. Overlaying a series of AP signals can be used to increase the number of data points on the fast depolarization phase. However, to increase accuracy for d*V*/d*t*_max_ estimation, a measurement technique with higher temporal-resolution (e.g., fiber optics; Ref. 45) would be preferable.

Besides cardiac tissue slices, the data analysis routine presented in this article will be applicable to other preparations, from single cell or cell cultures to in silico simulated and native tissue AP recordings, and help to bridge the gap between experimental and computational modeling of the heart (59). Certain adjustments of threshold values (e.g., to identify upstroke timing) will be required when adopting it to other preparations.

### Conclusions

Our data demonstrate that multiparametric optical mapping, here of *V*_m_ and CaT, can be conducted on cardiac tissue slices of rabbit and guinea pig LV. We provide a method for semiautomated data processing and for extracting important descriptors of AP (and CaT) shape and conduction properties.

The use of cardiac tissue slices would benefit from standardization, and we suggest adherence to the following points, or at least detailed documentation of equivalent aspects of the protocol:

*1*) Slices should be cut in an epicardium-tangential plane from electromechanically uncoupled tissue, using a high-precision vibratome with a low blade advance speed (∼0.03 mm/s), sectioning tissue at no more than ∼400 μm thickness;

*2*) Postcutting, a recovery period in warm solution is needed for slices, cut in ice-cold solution, to reach steady-state electrophysiological properties; the recovery time may be species dependent (∼35 min in guinea pig, ∼ 60 min in rabbit); and

*3*) Multiple-site point stimulation and field stimulation should be used for uncovering electrophysiological tissue heterogeneities, and any before/after investigations (such as drug testing) should control pacing location to exclude contributions of source-sink mismatches to the observed effects.

## GRANTS

This work was supported by the British Heart Foundation (grants to P. Kohl and C. Bollensdorff). We further gratefully acknowledge Microsoft Research (fellowship to K. Wang) and the University of Oxford Clarendon Fund (scholarship to P. Lee) and support from the Magdi Yacoub Institute and Qatar Foundation. G. R. Mirams and D. J. Gavaghan gratefully acknowledge support from an Engineering and Physical Sciences Research Council (EPSRC)/NC3Rs Strategic Award in Mathematics in Toxicology (NC/K001337/1) and the 2020 Science Programme funded through the EPSRC Cross-Discipline Interface Programme (EP/I017909/1). P. Kohl is a Senior Fellow of the British Heart Foundation and acknowledges support from the ERC Advanced Grant CardioNECT.

## DISCLOSURES

No conflicts of interest, financial or otherwise, are declared by the author(s).

## AUTHOR CONTRIBUTIONS

Author contributions: K.W., P.L., and C.B. conception and design of research; K.W., P.L., P.S., T.K.B., and C.B. performed experiments; K.W. and C.B. analyzed data; K.W., G.R.M., T.K.B., D.J.G., P.K., and C.B. interpreted results of experiments; K.W., T.K.B., and C.B. prepared figures; K.W., P.L., and C.B. drafted manuscript; K.W., P.L., G.R.M., P.S., T.K.B., D.J.G., P.K., and C.B. edited and revised manuscript; K.W., P.L., G.R.M., P.S., T.K.B., D.J.G., P.K., and C.B. approved final version of manuscript.

## Supplementary Material

Video S1
